# Parsing neurobiological heterogeneity of the clinical high-risk state for psychosis: A pseudo-continuous arterial spin labelling study

**DOI:** 10.3389/fpsyt.2023.1092213

**Published:** 2023-03-08

**Authors:** Dominic Oliver, Cathy Davies, Fernando Zelaya, Pierluigi Selvaggi, Andrea De Micheli, Ana Catalan, Helen Baldwin, Maite Arribas, Gemma Modinos, Nicolas A. Crossley, Paul Allen, Alice Egerton, Sameer Jauhar, Oliver D. Howes, Philip McGuire, Paolo Fusar-Poli

**Affiliations:** ^1^Early Psychosis: Interventions and Clinical-detection (EPIC) Lab, Department of Psychosis Studies, Institute of Psychiatry, Psychology and Neuroscience, King's College London, London, United Kingdom; ^2^Department of Psychiatry, University of Oxford, Oxford, United Kingdom; ^3^NIHR Oxford Health Biomedical Research Centre, Oxford, United Kingdom; ^4^Department of Psychosis Studies, Institute of Psychiatry, Psychology and Neuroscience, King's College London, London, United Kingdom; ^5^Department of Neuroimaging, Centre for Neuroimaging Sciences, Institute of Psychiatry, Psychology and Neuroscience, King’s College London, London, United Kingdom; ^6^Department of Translational Biomedicine and Neuroscience (DiBraiN), University of Bari Aldo Moro, Bari, Italy; ^7^OASIS Service, South London and Maudsley NHS Foundation Trust, London, United Kingdom; ^8^Mental Health Department, Basurto University Hospital, Facultad de Medicina y Odontología, Campus de Leioa, Biocruces Bizkaia Health Research Institute, UPV/EHU, University of the Basque Country, Barakaldo, Spain; ^9^NIHR Mental Health Policy Research Unit, Division of Psychiatry, University College London, London, United Kingdom; ^10^Department of Psychiatry, School of Medicine, Pontificia Universidad Católica de Chile, Santiago, Chile; ^11^Department of Psychology, University of Roehampton, London, United Kingdom; ^12^Department of Psychological Medicine, Institute of Psychiatry, Psychology and Neuroscience, King's College London, London, United Kingdom; ^13^Maudsley Biomedical Research Centre, South London and Maudsley NHS Foundation Trust, National Institute for Health Research, London, United Kingdom; ^14^Department of Brain and Behavioral Sciences, University of Pavia, Pavia, Italy

**Keywords:** clinical high risk for psychosis, brief limited intermittent psychotic symptoms, attenuated psychosis syndrome, arterial spin labelling, neuroimaging

## Abstract

**Introduction:**

The impact of the clinical high-risk for psychosis (CHR-P) construct is dependent on accurately predicting outcomes. Individuals with brief limited intermittent psychotic symptoms (BLIPS) have higher risk of developing a first episode of psychosis (FEP) compared to individuals with attenuated psychotic symptoms (APS). Supplementing subgroup stratification with information from candidate biomarkers based on neurobiological parameters, such as resting-state, regional cerebral blood flow (rCBF), may help refine risk estimates. Based on previous evidence, we hypothesized that individuals with BLIPS would exhibit increased rCBF compared to APS in key regions linked to dopaminergic pathways.

**Methods:**

Data from four studies were combined using ComBat (to account for between-study differences) to analyse rCBF in 150 age- and sex-matched subjects (*n* = 30 healthy controls [HCs], *n* = 80 APS, *n* = 20 BLIPS and *n* = 20 FEP). Global gray matter (GM) rCBF was examined in addition to region-of-interest (ROI) analyses in bilateral/left/right frontal cortex, hippocampus and striatum. Group differences were assessed using general linear models: (i) alone; (ii) with global GM rCBF as a covariate; (iii) with global GM rCBF and smoking status as covariates. Significance was set at *p* < 0.05.

**Results:**

Whole-brain voxel-wise analyses and Bayesian ROI analyses were also conducted. No significant group differences were found in global [*F*(3,143) = 1,41, *p* = 0.24], bilateral frontal cortex [*F*(3,143) = 1.01, *p* = 0.39], hippocampus [*F*(3,143) = 0.63, *p* = 0.60] or striatum [*F*(3,143) = 0.52, *p* = 0.57] rCBF. Similar null findings were observed in lateralized ROIs (*p* > 0.05). All results were robust to addition of covariates (*p* > 0.05). No significant clusters were identified in whole-brain voxel-wise analyses (*p* > 0.05_FWE_). Weak-to-moderate evidence was found for an absence of rCBF differences between APS and BLIPS in Bayesian ROI analyses.

**Conclusion:**

On this evidence, APS and BLIPS are unlikely to be neurobiologically distinct. Due to this and the weak-to-moderate evidence for the null hypothesis, future research should investigate larger samples of APS and BLIPS through collaboration across large-scale international consortia.

## Introduction

Psychotic disorders are prevalent, with an estimated 23.6 million cases worldwide, (1) and are associated with substantial clinical, personal, economic and societal burden (2). Under standard care, treatment is not initiated until the first episode of psychosis (FEP), which is associated with suboptimal clinical outcomes (2). Preventive approaches, such as intervening during the clinical high risk for psychosis (CHR-P) state (3), can alter the course of the disorder, improve long-term outcomes and reduce burden (2, 4).

To target interventions for CHR-P individuals at the highest risk of developing psychosis, accurate prediction of FEP onset is essential. Clinical interviews can be used to determine whether individuals meet CHR-P criteria, but while these have high specificity (i.e., adept at ruling out psychosis risk) their sensitivity is sub-optimal (i.e., many of those meeting CHR-P criteria will not develop psychosis) (5–7). For those who do meet CHR-P criteria, there is some refinement of prognostic risk prediction through the three CHR-P subgroups [attenuated psychotic symptoms (APS), brief limited intermittent psychotic symptoms (BLIPS) and genetic risk and deterioration (GRD)]. BLIPS is a relatively uncommon subgroup (10% of all CHR-P individuals) (8), where individuals experience a psychotic episode that spontaneously resolves within a week without antipsychotic treatment (9). BLIPS carry the highest level of psychosis risk (38% transition to psychosis within 2 years)(8) compared to APS (85% of CHR-P individuals; of whom 24% transition within 2 years) (8) and GRD (5% of CHR-P individuals; of whom 8% transition within 2 years) (8). However, it is currently not possible to stratify using clinical criteria beyond this, limiting the ability to accurately predict outcomes.

For more effective risk stratification, information is needed from additional sources, particularly from those directly relating to underlying neuropathology (i.e., biomarkers) as these will likely capture different elements of disorder progression. Alterations in frontal, hippocampal and striatal functioning reliably appear, persist and progress from the CHR-P state (10–15) to FEP (15–19) and chronic schizophrenia (13, 15, 20–22). Capturing these neurobiological changes could be informative in refining estimates of psychosis risk and enhancing risk stratification. Given the associated increase in psychosis risk estimates (23), we hypothesize that BLIPS may display more pronounced neurobiological differences compared to APS and GRD. However, there are currently no published studies investigating neurobiological differences between CHR-P subgroups due to the high prevalence of APS individuals in CHR-P samples.

Pseudo-continuous arterial spin labelling (ASL) allows for indirect measurement of regional, resting neuronal activity through regional cerebral blood flow (rCBF) (24), a potential biomarker for psychosis onset (10–12, 15). This approach exploits the intimate relationship between neuronal activity and its regional blood supply as a result of the phenomenon of neurovascular coupling. Modern pCASL sequences such as the one used in this study, are even more sensitive to neurovascular coupling as they can be tailored to reflect arterial blood flow from the vascular domain (arterioles and capillaries), where this phenomenon occurs.

CHR-P individuals display elevated dopamine signaling in the midbrain and striatum (25–28), as well as an altered relationship between hippocampal activation and striatal dopamine functioning (29, 30). This may in part stem from hippocampal gray matter abnormalities that have been consistently shown in CHR-P and FEP (31–34). CHR-P individuals display increased striatal rCBF and reduced frontal rCBF compared to clinical controls (15), with striatal rCBF correlating with attenuated positive symptom scores in CHR-P individuals (15). Similarly, CHR-P individuals have elevated rCBF in hippocampus and basal ganglia (10, 11), with hippocampal hyper-metabolism thought to drive downstream striatal dopamine dysfunction—a core mechanism underlying psychotic symptoms (13, 35, 36). There is evidence of hippocampal rCBF decreasing over time in CHR-P individuals who subsequently remitted from the CHR state (10). Additionally, there is a strong correlation between hippocampal rCBF and prefrontal GABA levels in CHR-P individuals who transition to psychosis, with no correlation in those who do not transition (12).

There are also distinct neurobiological differences between medicated and unmedicated FEP patients, with medicated patients showing increased rCBF in the striatum and reduced rCBF in the frontal cortex compared to clinical controls (15), while unmedicated FEP show reduced rCBF in the frontal cortex and no change in striatal rCBF compared to healthy controls (37).

Due to the low proportion of CHR-P individuals who meet BLIPS criteria in CHR-P samples, there has been difficulty in recruiting samples that are large enough to be sufficiently powered to investigate differences in rCBF between APS and BLIPS. Therefore, in this study, we combined data from four studies to investigate differences in global and regional rCBF between CHR-P subgroups. Due to the very small proportion of individuals meeting GRD criteria and this subgroup having psychosis risk that is not significantly different from HCs (8), analyses were restricted to APS and BLIPS subgroups. As rCBF in frontal, hippocampal and striatal regions appear to positively correlate with psychosis risk (10, 14, 24, 25), we expected rCBF in these regions in BLIPS individuals to be higher than in APS. As secondary hypotheses, we expected healthy controls (HCs) to have reduced rCBF in these regions compared to APS and BLIPS, with FEP patients having reduced rCBF compared to HCs, APS and BLIPS.

## Methods

### Participants

A total of 150 individuals were recruited across four studies conducted at the Institute of Psychiatry, Psychology & Neuroscience, King’s College London between 2008 and 2020 (Supplementary Figures S1, S2) (10, 37, 38). While several published papers have analyzed data that overlaps with our sample (10, 37, 38), the current combined analyses investigating the BLIPS subgroup, by means of newly available software designed for the combination of data from several studies, is new. Due to the relative low frequency of BLIPS compared to other groups, HC, APS and FEP individuals were age- and sex-matched to BLIPS participants.

HCs (*N* = 30) were recruited from the local community as part of three studies conducted at King’s College London (Supplementary Figure S1). Participants who were receiving prescription medications, had a history of psychiatric disorders, neurological illness or substance use disorder as specified in DSM-IV, were acutely intoxicated (assessed with alcohol breathalyser and urine drug screen) on the day of scanning, or had any contraindications to magnetic resonance imaging (MRI) were excluded from the study.

Help-seeking CHR-P individuals (APS and BLIPS; *N* = 100) aged 18–35 were recruited from four specialist early detection services: Outreach and Support in South London (OASIS) (39), Tower Hamlets Early Detection Service (THEDS), West London early intervention service and Cambridge shire and Peterborough Assessing Managing and Enhancing Outcomes (CAMEO). Both OASIS and THEDS are part of the Pan-London Network for Psychosis Prevention (PNP) (40). CHR-P status was determined using the Comprehensive Assessment of At-Risk Mental States (CAARMS) 12/2006 criteria (41). CHR-P subjects met criteria for either: (a) APS or, (b) BLIPS, (psychotic episode lasting < 1 week, remitting without treatment), both coupled with functional decline. Individuals were excluded if there was a history of previous psychotic disorder (with the exception of BLIPS, some of whom may meet acute and transient psychotic disorder criteria) (42) or manic episode, neurological disorder or current substance use disorder, estimated IQ < 70, acute intoxication (assessed with alcohol breathalyser and urine drug screen) on the day of scanning, and any contraindications to MRI. History of Axis I disorder(s) was not an exclusion criterion due to the transdiagnostic nature of the CHR-P state and the high prevalence of such diagnoses within these populations (43). CHR-P individuals were followed-up to assess potential transition to psychosis with the CAARMS, where possible, or using electronic health records.

FEP subjects (*n* = 20) were recruited from first episode psychosis teams within South London and Maudsley NHS Foundation Trust and Central and West London NHS Trust. To avoid the confounding effects of antipsychotics on CBF (44, 45), individuals recruited were antipsychotic naïve/free (where any prior antipsychotic treatment it was at most 4 weeks in total across the lifetime). Inclusion criteria for the FEP group were: psychotic disorder according to ICD-10 criteria; (46) in first episode of illness; no current antipsychotic treatment; and no antipsychotic treatment within the last 6 weeks for oral antipsychotics or 6 months for long-acting injectable antipsychotics (47, 48).

Ethical approval for all four studies was obtained from the National Health Service UK Research Ethics Committee, and all participants provided written informed consent to participate according to the Declaration of Helsinki.

### Design, materials, procedure

For descriptive purposes, we also collected information on medication history, tobacco and cannabis use, as well as global functioning using the Global Assessment of Functioning (GAF) (49).

### Magnetic resonance imaging acquisition and image processing

A total of 93 subjects (HCs *n* = 20, APS n = 59, BLIPS n = 14) were scanned with a General Electric Signa HDX 3 Tesla scanner (General Electric, Waukesha, Wisconsin, United States), using an 8-channel head coil as part of the NEUTOP and PROD studies (Supplementary Methods S1). A total of 27 subjects (APS *n* = 21, BLIPS *n* = 6) were scanned with a General Electric Discovery MR750 3 Tesla scanner (General Electric, Waukesha, Wisconsin, U) using a 32-channel head coil as part of the BRC-UHR study (Supplementary Methods S2). The remaining 30 subjects (HCs *n* = 10, FEP *n* = 20) were scanned with a General Electric Discovery MR750 3 Tesla scanner (General Electric, Waukesha, Wisconsin, United States), using a 12-channel head coil as part of the TR-FEP study (Supplementary Methods S3). In all cases, measurement of rCBF was carried out using a 3D pseudo-continuous Arterial Spin Labelling (3D-pCASL) sequence. This is the recommended method for CBF mapping by MRI, with an efficient four-pulse background suppression module to minimize static tissue signal, mitigating against any effects of subject motion (50). During data acquisition, participants were asked to maintain their gaze on a centrally-placed fixation cross. Full details of 3D-pCASL acquisition parameters and procedures are presented in Supplementary Methods S1–S3.

ASL data were pre-processed using the Automatic Software for ASL Processing (ASAP) 2.0 toolbox (51) running in Statistical Parametric Mapping version 12 (SPM12)^1^ and Matlab R2015b. (1) the origin of rCBF and 3D T1-weighted images were realigned; (2) 3D T1-weighted images were segmented using SPM segmentation to generate a binary mask including only brain tissues; (3) rCBF maps were co-registered to the corresponding 3D T1-weighted images; (4) a rough skull strip was performed on the rCBF map using FSL BET (52) (Brain Extraction Tool; −*f* = 0.4) to remove the majority of extra-cerebral signal with the rest removed by multiplication of the “brain only” binary mask, obtained in step 2, with the rCBF map in the space of the T1 image; and (5) T1-weighted scans and skull-stripped rCBF maps were spatially normalized to MNI avg152 standard space. Finally, rCBF maps were spatially smoothed using a 6 mm Gaussian smoothing kernel.

Because the rCBF data were acquired in four different studies across three scanners, ComBat^2^ (53–55) was used to harmonize the respective datasets across studies. The ComBat algorithm removes variation induced by scanner differences, while preserving between-subject biological variability through using an empirical Bayes framework (53–55). The algorithm estimates an empirical statistical distribution for the additive and multiplicative effects of scanner by assuming that all voxels share the same common distribution across ROIs, while maintaining the effects of biological covariates. Once this is calculated, the additive error terms can be derived and the data can be harmonized. Two advantages of this approach over other methods are that it improves the removal of scanner effects in datasets with small sample sizes, and does not make any assumptions about the neuroimaging technique being used (53, 56). This approach has been previously validated in the ENIGMA SCZ (57) and CHR-P (58) datasets. It has been shown that ComBat is superior to other harmonization techniques (e.g., global scaling, RAVEL) as it improves the replicability of voxels associated with biological covariates, improves statistical power, is robust to small sample sizes and recovers true effect sizes in imaging data (53). An important consideration is that ComBat can only maintain effects of measured and included biological covariates. If an important biological covariate is not measured or not included in the ComBat algorithm, this effect will not be preserved and may impact our ability to detect significant group differences. In order to preserve between-subject variability, the analysis included age, sex and group as covariates. The ComBat procedure was performed at an ROI-level for ROI analyses and at a voxel-wise level for voxel-wise analyses.

### Statistical analysis

#### Sociodemographic, clinical and substance use data

Differences between HC, APS, BLIPS and FEP were investigated using Fisher’s exact test for categorical variables due to small expected values in the contingency tables. General linear models were used for continuous variables to assess the effect of group on the variable of interest.

#### Global cerebral blood flow

To measure global rCBF signal, we used the ASAP toolbox to extract average rCBF values from a gray matter mask for each subject in standard space. The ICBM-152 mask was obtained from the DARTEL toolbox in SPM and thresholded to contain voxels with a > 0.25 probability of being gray matter. All subsequent analyses were conducted with and without global rCBF as covariate.

#### Region-of-interest analyses

Group effects on bilateral and lateralized frontal, hippocampal and striatal regional rCBF were determined using an ROI approach. ROIs were defined anatomically in MNI space using the cytoarchitectonic probabilistic atlas (59) as implemented in the Anatomy toolbox (60) in SPM. Mean regional rCBF values for the ROI were extracted for each subject using ASAP toolbox. Outliers were detected using Rosner’s test and removed. Outliers in rCBF values (2.52% of all values; *n* = 9 datapoints in HC; *n* = 17 APS; *n* = 0 BLIPS and *n* = 6 FEP) were replaced using multivariate imputation by chained equations (MICE) using the mice package (version 3.13.0) (61), following confirmation that values were missing completely at random (MCAR) using Little’s MCAR test.

Analyses of global gray matter and all ROIs (bilateral and lateralized frontal cortex, hippocampus and striatum) were conducted using general linear models to assess the effect of group on rCBF. All six contrasts (HC vs. APS, HC vs. BLIPS, HC vs. FEP, APS vs. BLIPS, APS vs. FEP, BLIPS vs. FEP) were of interest. As a second step, global rCBF smoking status, age and sex were added to the model as covariates (except for global gray matter rCBF, which just included smoking status, age and sex as covariates). As a third step, as antipsychotic medication is known to affect rCBF (44, 45), supplementary analyses were conducted with any subject with previous (i) antipsychotic exposure and (ii) antidepressant exposure removed from the model.

All ROI analyses were conducted using R version 3.6.3, with the “Emmeans” package (version 1.5.2–1) (62) used to calculate the estimated marginal mean contrasts. Alpha was set at *p* < 0.05.

#### Whole brain voxel-wise analyses

For completeness, we performed voxel-wise whole brain analyses in SPM12. Global gray matter rCBF was entered as a covariate. The following post-hoc contrasts were used: HC < APS; APS < BLIPS and BLIPS <FEP. Cluster-level inference was used (cluster forming threshold: *p* < 0.005 uncorrected); clusters were reported as significant at *p* < 0.05 using FWE correction in SPM, with analyses restricted to gray matter tissue using a binarized gray matter mask.

#### Bayesian region-of-interest analyses

Bayesian ROI analyses were conducted to improve understanding of potential null findings between APS and BLIPS groups. This approach allows for greater discrimination between “absence of evidence” and “evidence of absence” than frequentist statistics. Bayesian independent samples t-tests were used to compare rCBF in each ROI between APS and BLIPS. As our primary interest in these Bayesian analyses was to characterize potential null findings, an increase in Bayes factor (BF) in our analyses corresponds to an increase in evidence in favor of the null hypothesis (no differences in rCBF between APS and BLIPS). We used Jeffreys’ classification scheme (63) to interpret BF, with BF < 0.1 providing strong evidence for the alternative hypothesis; 0.1 < BF < 0.333 providing moderate evidence for an alternative hypothesis; 0.333 < BF < 1 providing weak evidence for an alternative hypothesis: 1 < BF < 3 providing weak evidence for the null hypothesis; 3 < BF < 10 providing moderate evidence for the null hypothesis; BF > 10 providing strong evidence for the null hypothesis. All Bayesian analyses were implemented in JASP (version 0.16.3), using the default uninformative priors from the software.

## Results

### Demographics

*N* = 30 HCs were recruited alongside *n* = 80 APS subjects, *n* = 20 BLIPS subjects and *n* = 20 FEP subjects. There were no significant differences in distribution of ethnicities between the groups (*p* = 0.47). The groups differed in clinical characteristics (Table 1). Psychotic, anxiety and depressive symptom severity was significantly higher in CHR-P subjects compared to HCs (*p* < 0.001). All groups were significantly different from each other in respect to global functioning (*p* < 0.001) with FEP subjects having the lowest GAF scores, followed by APS, BLIPS and HC, respectively.

**Table 1:** Participant sociodemographic, clinical and substance use characteristics.

		HC (*n* = 30)	APS (*n* = 80)	BLIPS (*n* = 20)	FEP (*n* = 20)	Statistic, *p*
		Mean (SD)	Mean (SD)	Mean (SD)	Mean (SD)	
**Sociodemographic**	Age, years	23.0 (4.08)	23.16 (4.37)	23.15 (4.48)	23.40 (4.06)	*F*(3,143) = 0.026, *p* = 0.99
	Sex; n (%)					*p* = 1
	*Male*	27 (90.0%)	72 (90.0%)	18 (90.0%)	18 (90.0%)	
	*Female*	3 (10.0%)	8 (10.0%)	2 (10.0%)	2 (10.0%)	
	Ethnicity; n (%)					*p* = 0.47
	*White*	18 (60.0%)	48 (60.0%)	10 (50.0%)	8 (40.0%)	
	*Black*	4 (13.3%)	14 (17.5%)	3 (15.0%)	5 (25.0%)	
	*Asian*	3 (10.0%)	4 (5.0%)	1 (5.0%)	3 (15.0%)	
	*Mixed*	2 (6.7%)	6 (7.5%)	1 (5.0%)	3 (15.0%)	
	*Missing*	3 (10.0%)	8 (10.0%)	5 (25.0%)	1 (5.0%)	
**Clinical**	CAARMS positive^a^	0.2 (0.76)	9.88 (3.96)	9.90 (5.22)	NA	*F*(2,110) = 50.45, *p* < 0.001
	PANSS positive	7.1 (0.32)	NA	NA	18.8 (6.86)	*F*(1,28) = 28.52, *p* < 0.001
	GAF	84.94 (11.39)	60.53 (10.91)	72.46 (13.51)	45.85 (14.29)	*F*(3,124) = 58.01, *p* < 0.001
	Previous antidepressant exposure; n (%)	0 (0.0%)	37 (46.0%)	2 (10.0%)	1 (5.0%)	*p* < 0.001
	Previous antipsychotic exposure; n (%)	0 (0.0%)	11 (13.8%)	1 (5.0%)	12 (60.0%)	*p* < 0.001
**Substance use**	Tobacco use, daily smoker; n (%)	6 (20%)	34 (42.5%)	7 (35.0%)	6 (30.0%)	*p* = 0.044
	Cannabis use, ever used; n (%)	17 (56.7%)	40 (50.0%)	7 (35.0%)	14 (70.0%)	*p* = 0.16
	*No use*	12 (40%)	32 (40.0%)	8 (40.0%)	NA	
	*Experimental use*	13 (43.3%)	10 (12.5%)	3 (15.0%)	NA	
	*Occasional use*	1 (3.3%)	13 (16.3%)	2 (10.0%)	NA	
	*Moderate use*	3 (10%)	14 (17.5%)	2 (10.0%)	NA	
	*Heavy use*	0 (0.0%)	3 (3.8%)	0 (0.0%)	NA	
	*Missing*	1 (3.3%)	8 (10.0%)	5 (25.0%)	NA	

a Sum of the global (severity) ratings for positive subscale items (P1-P4) of the CAARMS.

APS, attenuated psychotic symptoms; BLIPS, brief limited intermittent psychotic symptoms; CAARMS, comprehensive assessment of at risk mental states; FEP, first episode psychosis; GAF, global assessment of functioning; HC, healthy controls; PANSS, positive and negative syndrome scale.

*n* = 12 APS (15.0%) and *n* = 2 BLIPS (10.0%) were confirmed to have developed a psychotic disorder over follow-up (mean time to transition = 22.8 months; SD = 22.6). A higher proportion of APS subjects were daily tobacco smokers compared to HCs (*p* = 0.049). There were additionally significant differences in frequency of cannabis use between groups (*p* = 0.029). Sociodemographic, clinical and substance use descriptors are presented in Table 1. Percentage of missing values are presented in Supplementary Table S1.

### Global gray matter regional cerebral blood flow

**Figure 1:**
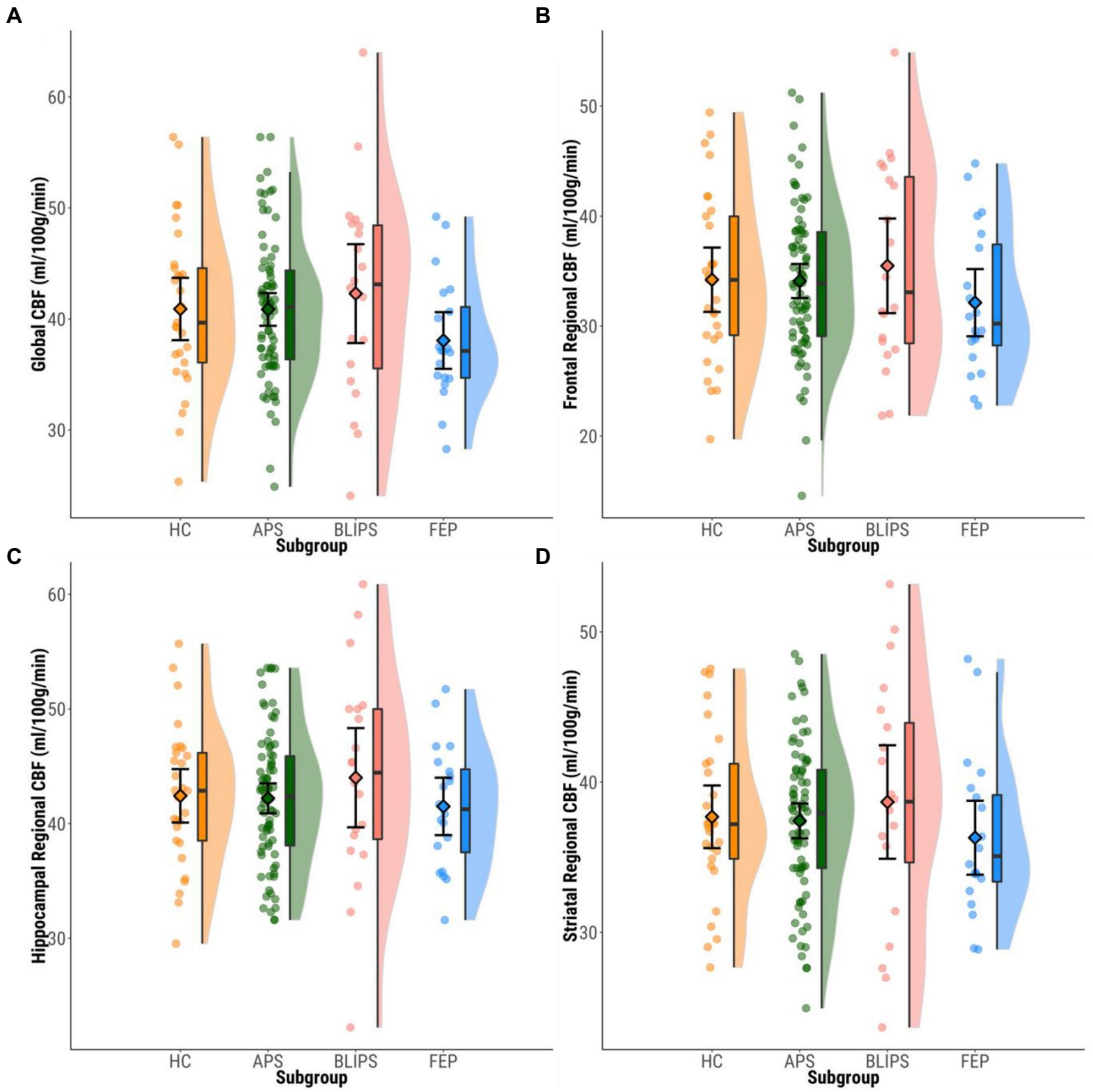
Plots showing mean bilateral ROI rCBF values within each group. Dots represent individual participants’ mean rCBF values. Boxplots show median values and interquartile ranges. Violin plots highlight the distribution of rCBF values within each group. Diamonds show group mean CBF values and 95%CIs. **(A)** Global CBF. **(B)** Frontal. **(C)** Hippocampus. **(D)** Striatum. CBF, cerebral blood flow; HC, healthy controls; APS, attenuated psychotic symptoms; BLIPS, brief limited intermittent psychotic symptoms; FEP, first episode psychosis.

No significant differences were found in global GM rCBF [*F*(3,143) = 1.408, *p* = 0.243] between HCs, APS, BLIPS, and FEP (Figure 1; Supplementary Table S2). These null findings were robust to the addition of smoking status as a covariate (*p* > 0.05; Supplementary Figure S3; Supplementary Tables S2–S4). There were additionally no differences seen in main models when individuals with previous antipsychotic exposure were excluded or when individuals with previous antidepressant exposure were excluded (*p* > 0.05, Supplementary Tables S11, S12).

### Region-of-interest regional cerebral blood flow

No significant differences were found in regional rCBF in bilateral frontal cortex [*F*(3,143) = 1.012, *p* = 0.389], bilateral hippocampus [*F*(3,143) = 0.625, *p* = 0.600], or bilateral striatum [*F*(3,143) = 0.522, *p* = 0.668] between HCs, APS, BLIPS and FEP (Figure 1; Supplementary Table S1). No significant differences were seen in regional rCBF in the left or right-lateralized frontal cortex, hippocampus or striatum (*p* > 0.05; Supplementary Tables S5, S8; Supplementary Figure S3).

These null findings were robust to the addition of (i) global rCBF alone; (ii) global rCBF, smoking status, age and sex as covariates (*p* > 0.05; Supplementary Figure S3; Supplementary Tables S2–S10). There were additionally no differences seen in main models when individuals with previous antipsychotic exposure (*n* = 11 APS; *n* = 1 BLIPS; *n* = 12 FEP) were excluded or when individuals with previous antidepressant exposure (*n* = 37 APS; *n* = 2 BLIPS; *n* = 1 FEP) were excluded (*p* > 0.05, Supplementary Tables S11, S12).

### Whole brain voxel-wise analyses

No significant group effects were identified in whole-brain rCBF analyses (p_FWE_ > 0.05).

### Bayesian region-of-interest analyses

**Figure 2:**
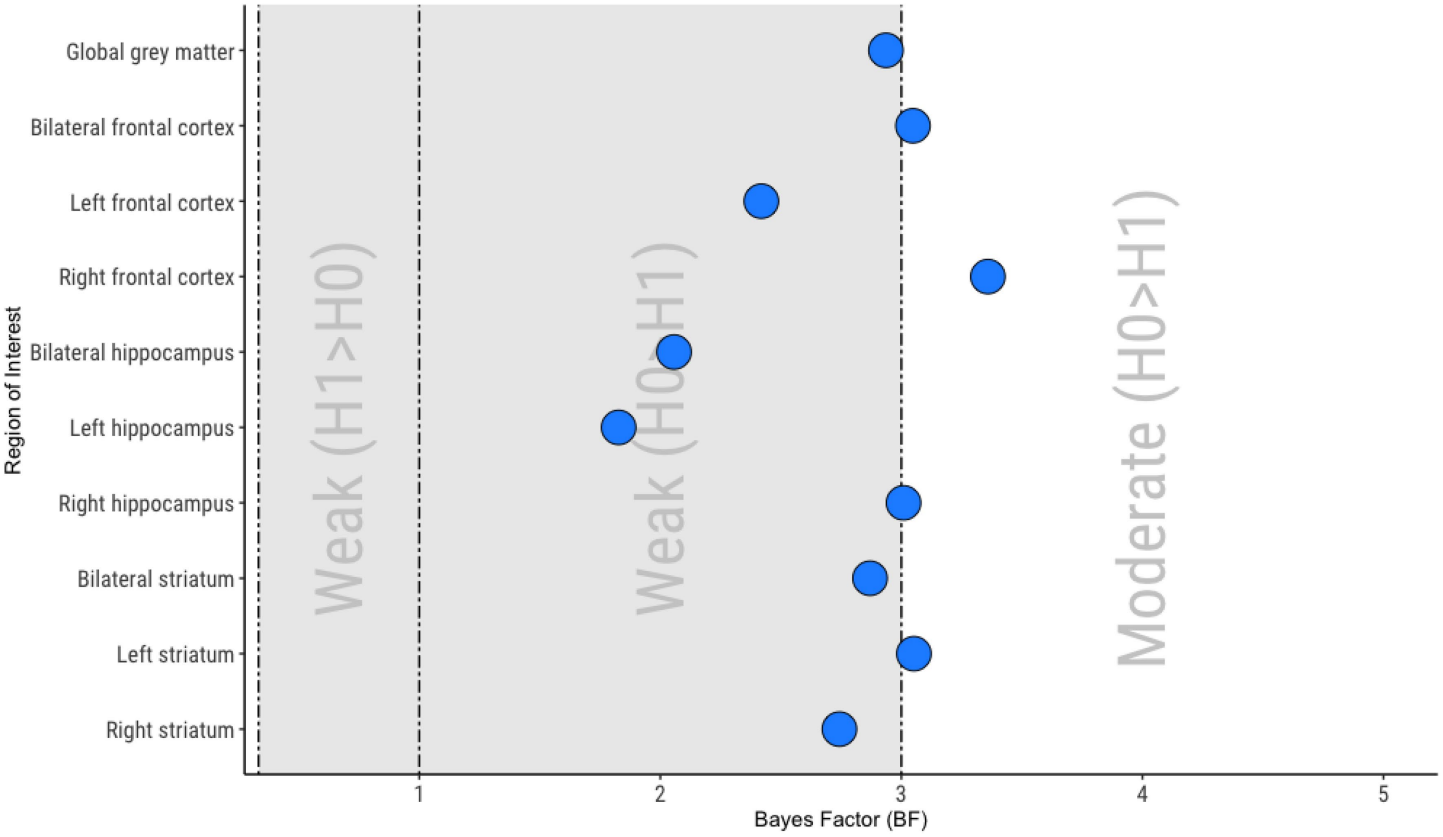
Summary of Bayesian analyses. 0.333 < Bayes Factor (BF) < 1, Weak evidence for the alternative hypothesis (H1; BLIPS rCBF > APS rCBF); 1 < BF < 3, Weak evidence for the null hypothesis (H0; no difference between APS rCBF and BLIPS rCBF); 3 < BF < 10, Moderate evidence for the null hypothesis (H0; no difference between APS rCBF and BLIPS rCBF).

Moderate support for the null hypothesis (no difference in rCBF between APS and BLIPS) was seen in bilateral frontal cortex (BF_01_ = 3.05), right frontal cortex (BF_01_ = 3.36), right hippocampus (BF_01_ = 3.01) and left striatum (BF_01_ = 3.05; Figure 2). Weak support for the null hypothesis was seen in global gray matter (BF_01_ = 2.94), left frontal cortex (BF_01_ = 2.42), bilateral hippocampus (BF_01_ = 2.10), left hippocampus (BF_01_ = 1.83), bilateral striatum (BF_01_ = 2.87) and right striatum (BF_01_ = 2.74; Figure 2).

## Discussion

To our knowledge, this is the largest neuroimaging dataset of CHR-P individuals meeting BLIPS criteria to date, and was achieved by combining data from four independently conducted studies. As such, this is the first study to compare rCBF between APS and BLIPS in order to investigate the potential of rCBF for risk stratification of CHR-P. However, our results do not provide evidence that rCBF, whether based on global gray matter, within ROIs strongly implicated in psychosis pathophysiology or within whole-brain voxel-wise analyses, is able to discriminate between BLIPS and APS at a group level. There are also no differences between these groups and HCs and FEP, in contrast to previous findings (10, 11, 37). In fact, these findings present moderate-to-weak evidence for no rCBF differences between APS and BLIPS across all ROIs.

Our results suggest that the APS and BLIPS CHR-P subgroups may not be neurobiologically distinct in terms of rCBF. However, the evidence for the null hypothesis ranges between ROIs from weak (e.g., left hippocampus) to moderate (e.g., right frontal cortex) so our findings may not be conclusive. The weakest evidence was for rCBF in the left hippocampus, which has been most strongly implicated in neurobiological contrasts between CHR-P and HCs (10–12). Previous research investigating neurobiological differences between subgroups of the CHR-P population have been mixed. For example longitudinal hippocampal rCBF changes are associated with remission from the CHR-P state (10) but baseline hippocampal rCBF does not appear to be associated with transition to psychosis (12). This may indicate that trajectories of rCBF may be more predictive of clinical outcomes, rather than baseline values alone. Similarly, assessing rCBF alone may not be sufficient for distinguishing CHR-P subgroups. For example, the interaction between prefrontal GABA levels and hippocampal rCBF is significantly different in CHR-P individuals who transition and those who do not (12). Data from additional time points or imaging modalities could allow for greater discrimination between CHR-P sub-populations, including APS and BLIPS.

Sociodemographic differences between our sample and those of previous studies may account for some differences in the results. As BLIPS is a less common CHR-P subgroup compared to APS, all matching between subgroups was in relation to the BLIPS sample. This means that the characteristics of HC, APS and FEP included in this study were selected to match the BLIPS cohort and are therefore not necessarily representative of their underlying populations. For example, despite the proportion of males historically being generally similar across APS and BLIPS (8, 23, 42, 64), due to one study (contributing n = 6 BLIPS; 30% of the total sample) only recruiting males, 90% of our BLIPS sample was male, which is substantially higher compared to previous studies (closer to 50%) (10–12, 15). While greater proportion of males is associated with greater psychosis risk in BLIPS samples (65), this is also the case in CHR-P more broadly (66). Enrichment of psychosis risk through other sociodemographic factors outside of CHR-P subgroup (e.g., migrant status, childhood trauma, urbanicity, parental severe mental illness, etc) (67) could have reduced the likelihood of finding evidence of neurobiological between-group differences and may reduce generalisability more broadly. Sex can similarly impact rCBF, with rCBF generally higher across all brain structures in females compared to males (68). By matching groups for age and sex, our analyses reduced the potential impact of sex, as well as age effects (68). While explicit matching has not been used in previous CHR-P research, age and sex have consistently been included in sensitivity analyses as covariates and significant differences between CHR-P and HCs in rCBF were retained (10–12, 15). Moreover, differences in rCBF remained non-significant when sex was included in the model, emphasizing that sex likely did not impact our results.

Two of the most robust predictors of psychosis onset in CHR-P individuals are attenuated positive psychotic symptom severity and global functioning at baseline, but the pattern of presentation varies between APS and BLIPS (66). The acute onset and spontaneous resolution of BLIPS frank psychotic symptoms (42) could have impacted rCBF levels, reducing the likelihood of finding differences between BLIPS and APS. While BLIPS experience positive psychotic symptoms at higher severity than the attenuated positive psychotic symptoms experienced by APS individuals, these are experienced for 1 week or less before spontaneously resolving (42). The BLIPS individuals were scanned after this period of frank psychotic symptoms had resolved, as indicated by the similarity of CAARMS scores between APS and BLIPS subjects. This is further emphasized by the significantly higher levels of functioning, as measured by the GAF, seen in BLIPS compared to APS subjects in this study. The dynamic nature of rCBF combined with its associations with symptom severity (69) and medication effects (44), which can also fluctuate, mean that scans performed at different times may result in altered perfusion. For example, greater differences may be seen in rCBF if participants were scanned at the peak of symptom severity, however, this would be ethically and logistically challenging. It also could be argued that despite remission from full psychosis that BLIPS still have a greater risk of transition compared to APS so finding neurobiological differences, such as rCBF, would still be plausible. In addition, many BLIPS individuals also experience attenuated psychotic symptoms, meeting criteria for both BLIPS and APS. Such individuals were considered to be solely BLIPS in these analyses. These factors may contribute to the observed similarities between APS and BLIPS in terms of rCBF.

Low rates of transition to FEP could also dilute potential differences in perfusion between APS and BLIPS subgroups. While expected transition rates are 24 and 38% in APS and BLIPS, respectively, (23) only 15% of APS individuals and 10% of BLIPS individuals in our sample developed a psychotic disorder over follow-up. Due to the demands of the study procedures, it may be that the recruited CHR-P individuals were less severely unwell than the CHR-P population average. The low transition rate may have reduced potential differences in rCBF between APS and BLIPS and limited our ability to explore any putative relationship between baseline rCBF and subsequent transition.

Despite the large overall sample, the power was still limited, particularly for the BLIPS subgroup. The literature base exploring rCBF in CHR-P individuals is not yet extensive with only a few papers published (10–12, 15). Due to this and the weak-to-moderate evidence for the null hypothesis and fluctuations in neurobiology over the timecourse of the CHR-P state, future research should aim to investigate larger samples of BLIPS and APS with multiple imaging modalities acquired at multiple time points, which can be achieved through collaboration and harmonization of data through large scale international consortia, e.g., HARMONY incorporating NAPLS (70), PRONIA (71), PSYSCAN (72), and ENIGMA (73). Similarly, recruitment of BLIPS could be improved through expanding CHR-P detection efforts to other brief psychotic conditions (e.g., acute and transient psychotic disorders and brief psychotic episodes), which all have significant overlap with BLIPS (65, 74–77). More consistent changes in cerebral perfusion in psychosis have been seen in studies measuring cerebral blood volume (CBV) compared to rCBF; (78) this may be a promising avenue to explore in future research. A potential limitation of our study may arise from the use of slightly different 3D pCASL acquisition protocols (see Supplementary Methods S1–S3). Since the data originate from multiple studies spanning several years, improvements made to the 3D pCASL pulse sequence allowed us to use a longer post-labelling delay in a relatively small number of the datasets acquired at a later stage (*n* = 27; 18%). Thus, there may be slightly different sensitivity to tissue perfusion in those data, although we used ComBat to mitigate against this. Moreover, due to a lack of harmonization between study protocols, we were limited in terms of available covariates (e.g., differences in instruments to assess anxiety meant we were unable to include this in statistical models, despite anxiety showing effects on rCBF in past research) (79, 80). However, the current analysis is focused on clinical prediction which needs methods that are robust to between-site differences in scanners, local methodologies, participant populations and other factors. Further to this, our groups were defined on the basis of investigating potential differences between BLIPS and the other groups. We therefore cannot conclude that there are no differences between all groups, only that we were unable to find a statistically significant difference between BLIPS and the other groups.

In conclusion, we have found weak-to-moderate evidence for an absence of difference in global GM, frontal, hippocampal and striatal rCBF between APS and BLIPS. Moreover, we did not find differences in rCBF between BLIPS and HCs or FEP either in whole-brain voxel-wise analysis or ROIs. These results suggest that rCBF alone may not be suitable for risk stratification in CHR-P individuals.

## Data availability statement

The datasets presented in this article are not readily available because at the time the data was collected it was not routine for participants to be asked for their consent to share data publicly, so this permission was not obtained. Whilst we are in favor of data being open access, no supporting data is available for this study. Requests to access the datasets should be directed to DO (dominic.a.oliver@kcl.ac.uk).

## Ethics statement

The studies involving human participants were reviewed and approved by National Health Service UK Research Ethics Committee. The patients/participants provided their written informed consent to participate in this study.

## Author contributions

PA, AE, PM, and PF-P: conceptualization. DO, CD, FZ, PS, AD, AC, HB, MA, and NC: data processing. DO, FZ, and PS: data analysis. DO: writing–original draft. All authors: interpretation and writing–review and editing. All authors contributed to the article and approved the submitted version.

## Funding

This study was supported by Wellcome Trust grants to PF-P (215793/Z/19/Z) and OH (094849/Z/10/Z), Medical Research Council (MRC) grants to PM (G0700995) and OH (MC_A656_5QD30_2135), a Maudsley Charity grant (No. 666) to OH, and the National Institute for Health Research (NIHR) Biomedical Research Centre (BRC) at South London and Maudsley NHS Foundation Trust and King’s College London (PM, OH, and PF-P).

## Conflict of interest

SJ has received honoraria for educational talks given for Lundbeck, Janssen, and Sunovion. OH has received investigator-initiated research funding from and/or participated in advisory/speaker meetings organized by Angelini, Autifony, Biogen, Boehringer-Ingelheim, Eli Lilly, Heptares, Global Medical Education, Invicro, Janssen, Lundbeck, Neurocrine, Otsuka, Sunovion, Recordati, Roche and Viatris/Mylan. OH has a patent for the use of dopaminergic imaging. OH is a part-time employee and stockholder of Lundbeck A/S. PF-P has received research funds or personal fees from Lundbeck, Angelini, Menarini, Sunovion, Boehringer Ingelheim, Mindstrong, Proxymm Science, outside the current study.

The remaining authors declare that the research was conducted in the absence of any commercial or financial relationships that could be construed as a potential conflict of interest.

## Publisher’s note

All claims expressed in this article are solely those of the authors and do not necessarily represent those of their affiliated organizations, or those of the publisher, the editors and the reviewers. Any product that may be evaluated in this article, or claim that may be made by its manufacturer, is not guaranteed or endorsed by the publisher.

## Footnotes

^1^ https://www.fil.ion.ucl.ac.uk/spm/

^2^ https://github.com/Jfortin1/ComBatHarmonization
