# Supplementary material for: Parsing neurobiological heterogeneity of the clinical high-risk state for psychosis: A pseudo-continuous arterial spin labelling study
Source: Front Psychiatry. 2023 Mar 8;14:1092213. doi: 10.3389/fpsyt.2023.1092213 (PMC10031088; doi:10.3389/fpsyt.2023.1092213)
Supplement: Supplementary file 1 [file Data_Sheet_1.docx]

**Supplementary Material**

Oliver D, Davies C, Zelaya F et al. Parsing neurobiological heterogeneity of the clinical high risk state for psychosis: a pseudo-continuous arterial spin labelling study

**Supplementary Figure 1** Summary of study participants in each group

**Supplementary Figure 2** Summary of proportion of data included in analysis following age- and gender-matching compared to total available data stratified by group

**Supplementary Methods S1** MRI acquisition protocol 1

**Supplementary Methods S2** MRI acquisition protocol 2

**Supplementary Methods S3** MRI acquisition protocol 3

**Supplementary Figure 3** Left- and right-lateralised rCBF across ROIs

**Supplementary Table S1** Proportion of missing data

**Supplementary Table S2** Results of bilateral ROI general linear models (no covariates)

**Supplementary Table S3** Results of bilateral ROI general linear models including global CBF as a covariate

**Supplementary Table S4** Results of bilateral ROI general linear models including covariates

**Supplementary Table S5** Results of left-lateralised ROI general linear models (no covariates)

**Supplementary Table S6** Results of left-lateralised ROI general linear models including global CBF as a covariate

**Supplementary Table S7** Results of left-lateralised ROI general linear models including covariates

**Supplementary Table S8** Results of right-lateralised ROI general linear models (no covariates)

**Supplementary Table S9** Results of right-lateralised ROI general linear models including global CBF as a covariate

**Supplementary Table S10** Results of right-lateralised ROI general linear models including covariates

**Supplementary Table S11** Results of ROI general linear models excluding subjects with prior antipsychotic exposure

**Supplementary Table S12** Results of ROI general linear models excluding subjects with prior antidepressant exposure

**Supplementary References**

**
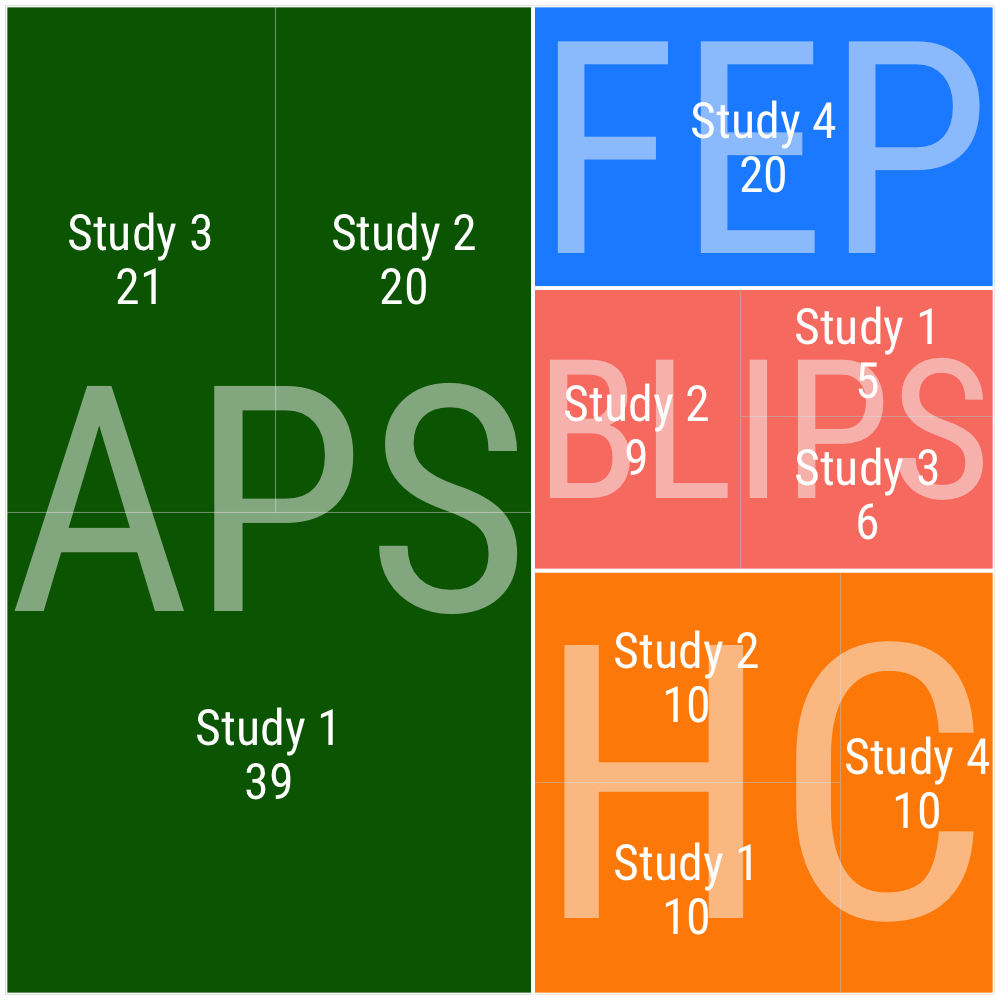
Supplementary Figure 1** Summary of study participants in each group

**
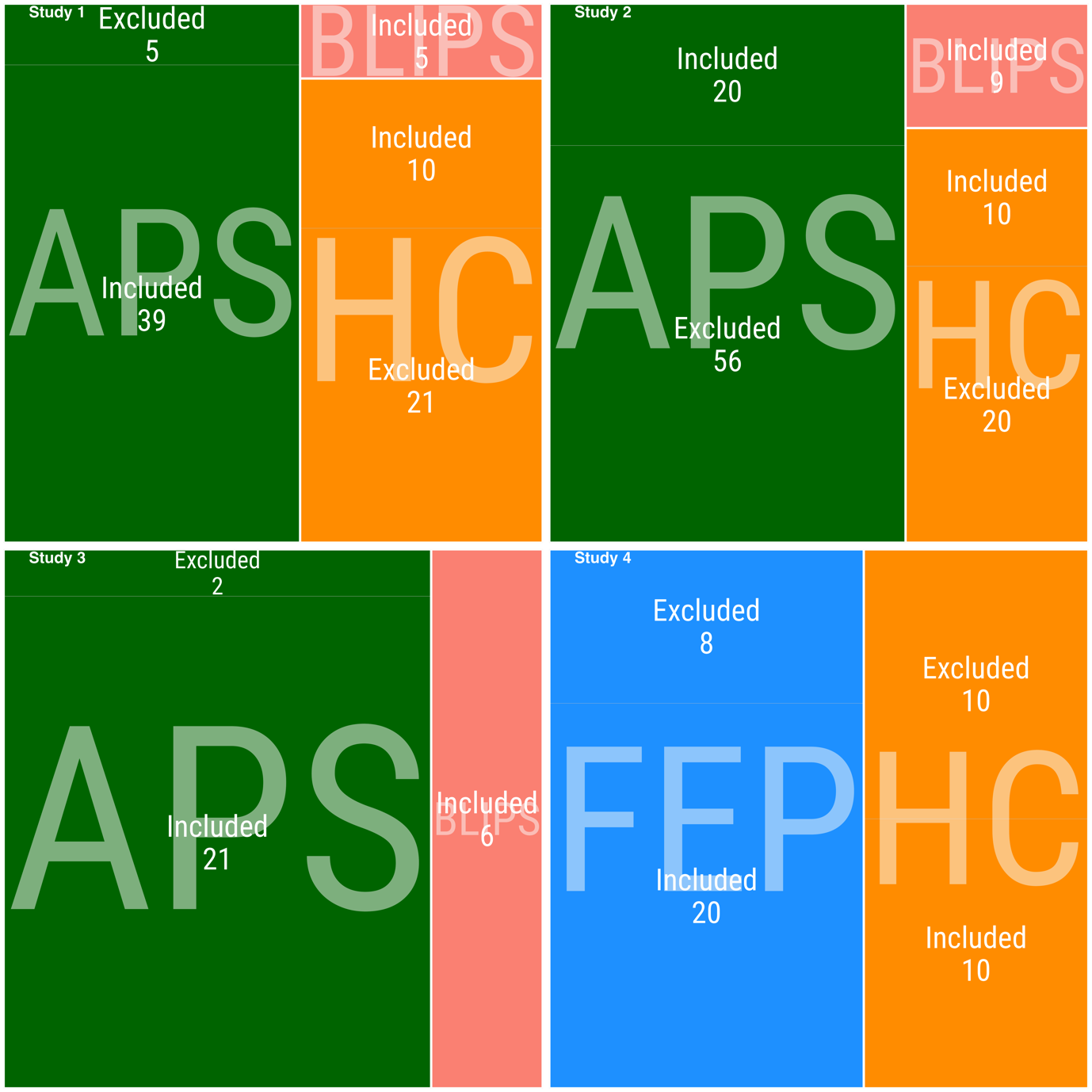
Supplementary Figure 2** Summary of proportion of data included in analysis following age- and gender-matching compared to total available data stratified by group

**Supplementary Methods S1** MRI acquisition protocol 1 (Study 1, Study 2; adapted from ^1^)

103 subjects (HC n=30, APS n=59, BLIPS n=14) were scanned with their eyes open using a General Electric Signa HDX 3 Tesla scanner (General Electric, Chicago, USA), fitted with a receive only 8-channel phased array head. For image registration a high-resolution T1-weighted Spoiled Gradient Recalled (SPGR) image (1.1x1.1x1.1mm, TE=2.848, TR=7.144ms, Flip angle=20deg, FoV=280) was acquired. Resting Cerebral Blood Flow (rCBF) was measured using Continuous Arterial Spin Labelling (CASL) scans acquired with a 3D Fast Spin Echo (FSE), coronal multi-shot readout, following a post-labelling delay of 1.5s. The spiral acquisition used a short (4ms) TE, and 8 spiral arms (interleaves) with 512 points in each arm. (FSE TE 32ms/TR = 5500ms; ETL = 64). Images were reconstructed to a 256 x 256 matrix, giving a final spatial resolution of 1x1 mm in plane. 60 slices of 3mm thickness were obtained. Three pairs of tagged-untagged images were collected. Background suppression included selective saturation of the image slab at 4.3s before acquisition, selective inversion 3s before acquisition and non-selective inversions at 1.5s, 764ms, 334ms and 84ms before imaging. This repeated inversion achieved successful suppression of the background static tissue signal, maximizing the sensitivity to blood perfusion. Calibration images were collected with the same imaging sequence but with inversion recovery preparation instead of CASL. One sequence with saturation of 4.3s and then an inversion at 1650 ms before imaging was used to create a fluid suppressed image. A second sequence with saturation at 4.3s and then inversion at both 2408ms and 511ms was also acquired to create a fluid and white matter suppressed image. For both these sequences, the receiver gain was automatically lowered by 21 dB relative to the ASL sequence to avoid receiver saturation. These images were used to quantify blood flow in physiological units (ml blood/100gm tissue/min). The sensitivity of the image to water was calibrated at each voxel 1-3. Using a neighborhood maximum algorithm to avoid regions with partial volume of suppressed fluid, a low resolution sensitivity map was created. This map was calibrated for water sensitivity by assuming the tissue was white matter with a water concentration of 0.735 gm/ml 4 and a T1 of 900ms, and using the equations for inversion recovery signal attenuation. By assuming gray matter with a water concentration of 0.88 gm/ml and a T1 of 1150 there was only a 5% calibration difference. This calibration produced a sensitivity map, C, equal to the fully relaxed MRI signal intensity produced by 1gm of water per ml of brain tissue. With this co-registered sensitivity map C, cerebral blood flow (CBF) was calculated. The whole ASL pulse sequence, including the acquisition of calibration images, was performed in 6:08min.

**Supplementary Methods S2** MRI acquisition protocol 2 (Study 3; adapted from ^2^)

27 subjects (APS n=21, BLIPS n=6) were scanned with a General Electric Discovery MR750 3 Tesla scanner (General Electric, Chicago, USA) using a 32-channel head coil. Measurement of Cerebral Blood Flow (CBF) was carried out using a 3D pseudo-continuous Arterial Spin Labelling (3D-pCASL) sequence. Labelling of arterial blood was achieved with a 1525ms train of Hanning-shaped radio frequency pulses in the presence of a net magnetic field gradient along the flow direction (the z-axis of the magnet). After a post-labelling delay of 2025ms, a whole brain volume was read using a 3D inter-leaved “stack-of-spirals” Fast Spin Echo readout,^3^ consisting of 8 interleaved spiral arms in the in-plane direction, with 512 points per spiral interleave. TE=11ms, TR=5135ms, and 56 slice-partitions of 3mm thickness were defined in the 3D readout. The in-plane FoV was 240×240mm. The spiral sampling of k-space was re-gridded to a rectangular matrix with an approximate in-plane resolution of 2 x 2 mm. The sequence used 4 control-label pairs. CBF maps were computed from the mean perfusion weighted difference image derived from the four control-label pairs, by scaling the difference image against a proton density image acquired at the end of the sequence, using identical readout parameters. This computation was done according to the formula suggested by the ISMRM perfusion study group and the European consortium for ASL in dementia.^4^ The sequence used four background suppression pulses to minimise static tissue signal at the time of image acquisition. The entire acquisition time of the 3D-pCASL sequence was 6:20 minutes. We also acquired a three-dimensional sagittal high-spatial-resolution Inversion Recovery Spoiled Gradient Echo (IR-SPGR) T1-weighted scan (TE=3.016ms, TR=7.31ms, TI=400ms, FoV=270mm). The final resolution of the image was 1.1 x 1.1 x 1.2mm.

**Supplementary Methods S3** MRI acquisition protocol 3 (Study 4; adapted from ^5^)

30 subjects (n=10 HC, n=20 FEP) were scanned with a GE MR750 3-T scanner and a 12-channel head coil. A T1-weighted MPRAGE scan was also acquired (FOV = 260 mm; echo time = 2.8 ms; repetition time = 6.98 ms; 256 × 256 matrix; slice thickness = 1.2 mm, flip angle = 11) for normalization purposes. A T2-weighted image (FOV = 240 mm; echo time = 54.68 ms; repetition time = 4380 ms; 320 × 320 matrix; slice thickness = 4 mm) was also acquired and used for the pre-processing of ASL images. ASL data were acquired using a pseudo-continuous arterial spin labelling sequence (PCASL). Four control-label pairs were used (labelling time = 1525 ms; post labelling delay = 1500 ms). Images were read with a 3D Fast-Spin echo stack of spirals scheme, consisting of eight inter-leaved spiral arms, 512 points per arm and 60 slice-locations of 3 mm thickness. The raw spatial resolution of the perfusion sensitive images was approximately 3.6 mm in plane and 3 mm through plane; and the data points were re-gridded to a rectangular matrix prior to Fourier transformation and written with a voxel size of 1 × 1 × 3 mm (no gap). The sequence uses four background suppression pulses to minimise static tissue signal at the time of image acquisition. Therefore, only four pairs of control-labelled images are required to produce reliable perfusion induced signal differences. The mean perfusion weighted difference image was derived from the average of the difference of the four Control-Label pairs.  Computation of voxel-wise CBF was achieved by dividing this mean difference image by a proton density image acquired at the end of the sequence, using identical readout parameters.  The entire acquisition time of the 3D-pCASL sequence was 6:08min


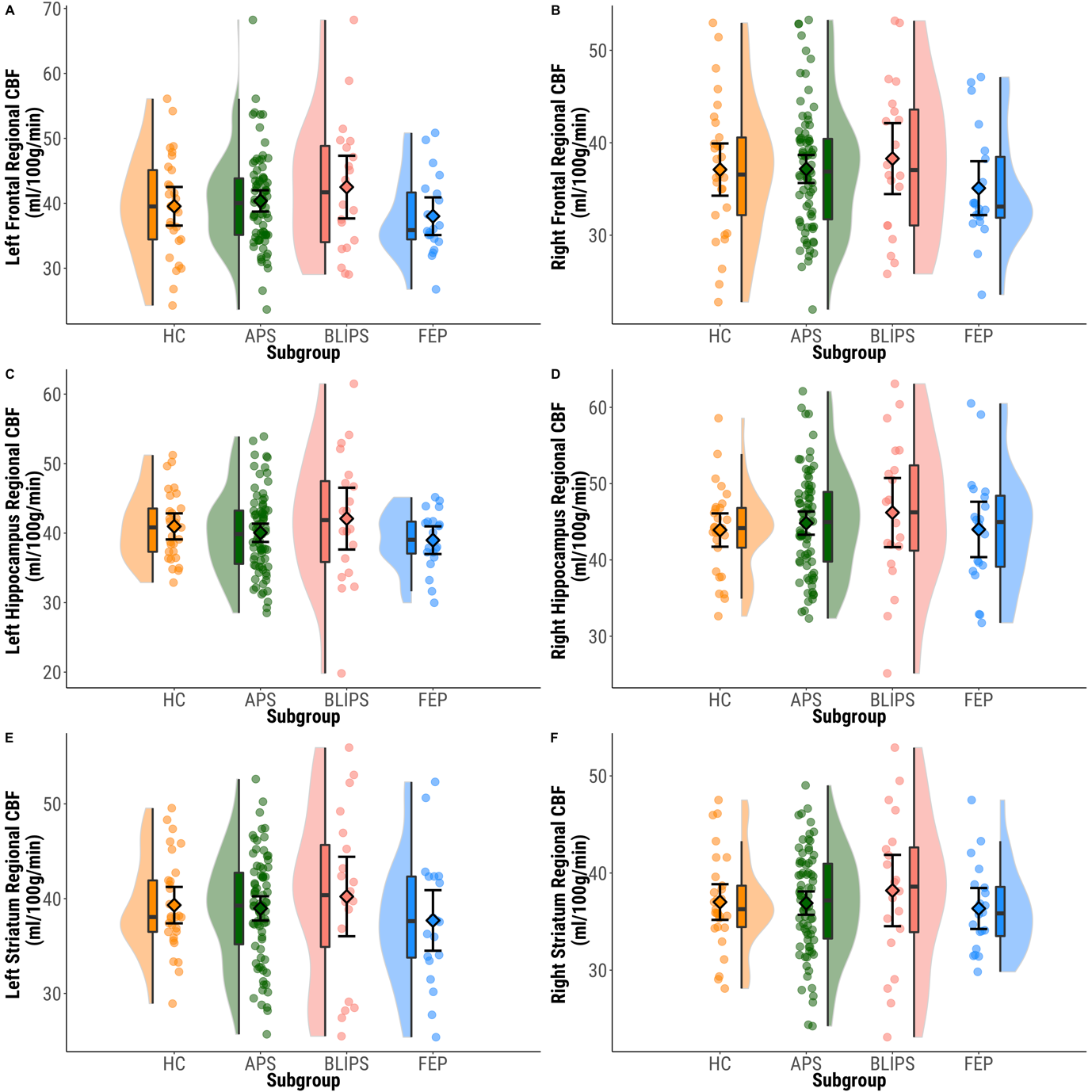
**Supplementary Figure 3** Plots showing mean left- and right-lateralised ROI rCBF values within each group. Dots represent individual participants’ mean rCBF values. Boxplots show median values and interquartile ranges. Violin plots highlight the distribution of rCBF values within each group. Diamonds show group mean CBF values and 95%CIs. **A** Left Frontal; **B** Right Frontal; **C** Left Hippocampus; **D** Right Hippocampus; **E** Left Striatum; **F** Right Striatum. CBF: cerebral blood flow; HC: healthy controls; APS: attenuated psychotic symptoms; BLIPS: brief limited intermittent psychotic symptoms; FEP: first episode psychosis.

| **Supplementary Table S1** Proportion of missing data in participant sociodemographic, clinical and substance use data | | | | | |  | |
| --- | --- | --- | --- | --- | --- | --- | --- |
|  |  | **HC (n=30)**  n (%) | **APS (n=80)**  n (%) | **BLIPS (n=20)**  n (%) | **FEP (n=20)**  n (%) | |  |
| **Sociodemographic** | Age | 0 (0.0%) | 0 (0.0%) | 0 (0.0%) | 0 (0.0%) | |  |
|  | Gender | 0 (0.0%) | 0 (0.0%) | 0 (0.0%) | 0 (0.0%) | |  |
|  | Ethnicity | 3 (10.0%) | 8 (10.0%) | 5 (25.0%) | 1 (5.0%) | |  |
| **Clinical** | CAARMS positive | 10 (33.3%) | 4 (5.1%) | 0 (0.0%) | 20 (100.0%) | |  |
|  | PANSS positive | 20 (66.7%) | 80 (100.0%) | 20 (100.0%) | 0 (0%) | |  |
|  | GAF | 3 (10.0%) | 10 (12.9%) | 6 (30.0%) | 0 (0%) | |  |
|  | Previous antidepressant exposure | 0 (0.0%) | 2 (2.6%) | 0 (0.0%) | 0 (0.0%) | |  |
|  | Previous antipsychotic exposure | 0 (0.0%) | 0 (100.0%) | 0 (0.0%) | 20 (0.0%) | |  |
| **Substance use** | Tobacco use, daily smoker | 0 (0.0%) | 0 (0.0%) | 0 (0.0%) | 0 (0.0%) | |  |
|  | Cannabis use, ever used | 11 (36.7%) | 8 (10.0%) | 5 (25.0%) | 20 (100.0%) | |  |

**Supplementary Table S1** Results of bilateral ROI general linear models (no covariates)

| **Contrast** | **Estimated marginal mean difference** | **Lower 95% CI** | **Upper 95% CI** | **p-value** |
| --- | --- | --- | --- | --- |
| **Global CBF** |  |  |  |  |
| HC - APS | 0.440 | -2.544 | 3.425 | 0.991 |
| HC - BLIPS | -0.090 | -4.077 | 3.898 | 1 |
| HC - FEP | 2.414 | -1.574 | 6.401 | 0.616 |
| APS - BLIPS | -0.530 | -3.969 | 2.909 | 0.99 |
| APS - FEP | 1.973 | -1.466 | 5.412 | 0.656 |
| BLIPS - FEP | 2.503 | -1.836 | 6.842 | 0.652 |
| **Frontal cortex** | | | | |
| HC - APS | 0.402 | -2.651 | 3.456 | 0.993 |
| HC - BLIPS | -0.213 | -4.293 | 3.867 | 1 |
| HC - FEP | 1.608 | -2.472 | 5.688 | 0.857 |
| APS - BLIPS | -0.616 | -4.134 | 2.903 | 0.985 |
| APS - FEP | 1.205 | -2.313 | 4.724 | 0.901 |
| BLIPS - FEP | 1.821 | -2.618 | 6.260 | 0.842 |
| **Hippocampus** | | | | |
| HC - APS | 0.316 | -2.378 | 3.010 | 0.995 |
| HC - BLIPS | -0.661 | -4.261 | 2.940 | 0.983 |
| HC - FEP | 0.248 | -3.352 | 3.848 | 0.999 |
| APS - BLIPS | -0.976 | -4.081 | 2.128 | 0.921 |
| APS - FEP | -0.068 | -3.172 | 3.037 | 1 |
| BLIPS - FEP | 0.909 | -3.008 | 4.826 | 0.966 |
| **Striatum** | | | | |
| HC - APS | 0.380 | -2.051 | 2.811 | 0.989 |
| HC - BLIPS | -0.238 | -3.487 | 3.011 | 0.999 |
| HC - FEP | 0.932 | -2.317 | 4.180 | 0.939 |
| APS - BLIPS | -0.618 | -3.419 | 2.184 | 0.971 |
| APS - FEP | 0.552 | -2.250 | 3.354 | 0.979 |
| BLIPS - FEP | 1.170 | -2.365 | 4.704 | 0.909 |

**Supplementary Table S2** Results of bilateral ROI general linear models with global CBF as a covariate

| **Contrast** | **Estimated marginal mean difference** | **Lower 95% CI** | **Upper 95% CI** | **p-value** |
| --- | --- | --- | --- | --- |
| **Frontal cortex** | | | | |
| HC - APS | 0.031 | -1.702 | 1.763 | 1 |
| HC - BLIPS | -0.138 | -2.452 | 2.177 | 0.999 |
| HC - FEP | -0.429 | -2.756 | 1.897 | 0.982 |
| APS - BLIPS | -0.168 | -2.165 | 1.828 | 0.998 |
| APS - FEP | -0.460 | -2.465 | 1.545 | 0.967 |
| BLIPS - FEP | -0.292 | -2.821 | 2.238 | 0.996 |
| **Hippocampus** | | | | |
| HC - APS | 0.032 | -1.862 | 1.926 | 1 |
| HC - BLIPS | -0.603 | -3.134 | 1.928 | 0.963 |
| HC - FEP | -1.307 | -3.851 | 1.237 | 0.729 |
| APS - BLIPS | -0.635 | -2.818 | 1.548 | 0.936 |
| APS - FEP | -1.339 | -3.531 | 0.854 | 0.609 |
| BLIPS - FEP | -0.704 | -3.470 | 2.063 | 0.956 |
| **Striatum** | | | | |
| HC - APS | 0.120 | -1.562 | 1.801 | 0.999 |
| HC - BLIPS | -0.185 | -2.431 | 2.061 | 0.998 |
| HC - FEP | -0.493 | -2.751 | 1.764 | 0.971 |
| APS - BLIPS | -0.305 | -2.242 | 1.633 | 0.989 |
| APS - FEP | -0.613 | -2.559 | 1.333 | 0.921 |
| BLIPS - FEP | -0.308 | -2.763 | 2.147 | 0.994 |

Abbreviations: APS, attenuated psychosis syndrome; BLIPS, brief limited intermittent psychosis syndrome; CBF, cerebral blood flow; CI, confidence interval; FEP, first episode psychosis; HC, healthy controls.

**Supplementary Table S3** Results of bilateral ROI general linear models with global CBF (for frontal cortex, hippocampus and striatum only), smoking status, age and sex as covariates

| **Contrast** | **Estimated marginal mean difference** | **Lower 95% CI** | **Upper 95% CI** | **p-value** |
| --- | --- | --- | --- | --- |
| **Global CBF** |  |  |  |  |
| HC - APS | 0.518 | -2.487 | 3.523 | 0.986 |
| HC - BLIPS | -0.088 | -4.094 | 3.918 | 1 |
| HC - FEP | 2.495 | -1.722 | 6.711 | 0.633 |
| APS - BLIPS | -0.606 | -4.049 | 2.838 | 0.985 |
| APS - FEP | 1.977 | -1.972 | 5.925 | 0.745 |
| BLIPS - FEP | 2.583 | -2.180 | 7.345 | 0.695 |
| **Frontal cortex** | | | | |
| HC - APS | -0.042 | -1.788 | 1.704 | 1 |
| HC - BLIPS | -0.451 | -2.777 | 1.875 | 0.98 |
| HC - FEP | -0.097 | -2.558 | 2.365 | 1 |
| APS - BLIPS | -0.409 | -2.409 | 1.592 | 0.976 |
| APS - FEP | -0.054 | -2.356 | 2.247 | 1 |
| BLIPS - FEP | 0.354 | -2.423 | 3.132 | 0.994 |
| **Hippocampus** | | | | |
| HC - APS | -0.075 | -2.006 | 1.855 | 1 |
| HC - BLIPS | -0.613 | -3.186 | 1.959 | 0.963 |
| HC - FEP | -1.284 | -4.006 | 1.438 | 0.778 |
| APS - BLIPS | -0.538 | -2.750 | 1.674 | 0.961 |
| APS - FEP | -1.209 | -3.753 | 1.336 | 0.774 |
| BLIPS - FEP | -0.671 | -3.742 | 2.401 | 0.971 |
| **Striatum** | | | | |
| HC - APS | 0.111 | -1.582 | 1.804 | 0.999 |
| HC - BLIPS | -0.384 | -2.640 | 1.872 | 0.986 |
| HC - FEP | -0.440 | -2.827 | 1.947 | 0.982 |
| APS - BLIPS | -0.495 | -2.435 | 1.445 | 0.956 |
| APS - FEP | -0.550 | -2.782 | 1.681 | 0.96 |
| BLIPS - FEP | -0.056 | -2.749 | 2.638 | 1 |

Abbreviations: APS, attenuated psychosis syndrome; BLIPS, brief limited intermittent psychosis syndrome; CBF, cerebral blood flow; CI, confidence interval; FEP, first episode psychosis; HC, healthy controls.

**Supplementary Table S4** Results of left lateralised ROI general linear models (no covariates).

| **Contrast** | **Estimated marginal mean difference** | **Lower 95% CI** | **Upper 95% CI** | **p-value** |
| --- | --- | --- | --- | --- |
| **Frontal cortex** |  |  |  |  |
| HC - APS | -0.181 | -3.308 | 2.947 | 0.999 |
| HC - BLIPS | -1.310 | -5.490 | 2.870 | 0.922 |
| HC - FEP | 0.920 | -3.260 | 5.100 | 0.971 |
| APS - BLIPS | -1.130 | -4.734 | 2.475 | 0.922 |
| APS - FEP | 1.100 | -2.504 | 4.705 | 0.927 |
| BLIPS - FEP | 2.230 | -2.318 | 6.778 | 0.757 |
| **Hippocampus** |  |  |  |  |
| HC - APS | 1.055 | -1.540 | 3.650 | 0.845 |
| HC - BLIPS | -0.049 | -3.517 | 3.420 | 1 |
| HC - FEP | 1.457 | -2.011 | 4.925 | 0.832 |
| APS - BLIPS | -1.104 | -4.095 | 1.887 | 0.879 |
| APS - FEP | 0.402 | -2.589 | 3.393 | 0.993 |
| BLIPS - FEP | 1.506 | -2.268 | 5.279 | 0.853 |
| **Striatum** |  |  |  |  |
| HC - APS | 0.562 | -2.121 | 3.245 | 0.975 |
| HC - BLIPS | -0.020 | -3.605 | 3.566 | 1 |
| HC - FEP | 1.290 | -2.295 | 4.875 | 0.887 |
| APS - BLIPS | -0.582 | -3.674 | 2.510 | 0.981 |
| APS - FEP | 0.728 | -2.364 | 3.820 | 0.965 |
| BLIPS - FEP | 1.310 | -2.591 | 5.210 | 0.906 |

Abbreviations: APS, attenuated psychosis syndrome; BLIPS, brief limited intermittent psychosis syndrome; CBF, cerebral blood flow; CI, confidence interval; FEP, first episode psychosis; HC, healthy controls.

**Supplementary Table S5** Results of left lateralised ROI general linear models with global CBF as a covariate.

| **Contrast** | **Estimated marginal mean difference** | **Lower 95% CI** | **Upper 95% CI** | **p-value** |
| --- | --- | --- | --- | --- |
| **Frontal cortex** |  |  |  |  |
| HC - APS | -0.606 | -1.826 | 0.614 | 0.749 |
| HC - BLIPS | -1.224 | -2.854 | 0.406 | 0.433 |
| HC - FEP | -1.411 | -3.050 | 0.227 | 0.309 |
| APS - BLIPS | -0.618 | -2.024 | 0.788 | 0.813 |
| APS - FEP | -0.805 | -2.217 | 0.607 | 0.66 |
| BLIPS - FEP | -0.188 | -1.969 | 1.594 | 0.997 |
| **Hippocampus** |  |  |  |  |
| HC - APS | 0.805 | -1.167 | 2.776 | 0.844 |
| HC - BLIPS | 0.002 | -2.631 | 2.636 | 1 |
| HC - FEP | 0.085 | -2.562 | 2.732 | 1 |
| APS - BLIPS | -0.802 | -3.074 | 1.469 | 0.892 |
| APS - FEP | -0.720 | -3.002 | 1.562 | 0.92 |
| BLIPS - FEP | 0.082 | -2.796 | 2.961 | 1 |
| **Striatum** |  |  |  |  |
| HC - APS | 0.276 | -1.585 | 2.137 | 0.991 |
| HC - BLIPS | 0.038 | -2.448 | 2.525 | 1 |
| HC - FEP | -0.278 | -2.778 | 2.221 | 0.996 |
| APS - BLIPS | -0.237 | -2.382 | 1.908 | 0.996 |
| APS - FEP | -0.554 | -2.708 | 1.600 | 0.955 |
| BLIPS - FEP | -0.317 | -3.035 | 2.401 | 0.995 |

Abbreviations: APS, attenuated psychosis syndrome; BLIPS, brief limited intermittent psychosis syndrome; CBF, cerebral blood flow; CI, confidence interval; FEP, first episode psychosis; HC, healthy controls.

**Supplementary Table S6** Results of left lateralised ROI general linear models with global CBF, smoking status, age and sex as covariates.

| **Contrast** | **Estimated marginal mean difference** | **Lower 95% CI** | **Upper 95% CI** | **p-value** |
| --- | --- | --- | --- | --- |
| **Frontal cortex** |  |  |  |  |
| HC - APS | -0.570 | -1.811 | 0.671 | 0.792 |
| HC - BLIPS | -1.320 | -2.973 | 0.334 | 0.378 |
| HC - FEP | -1.296 | -3.045 | 0.453 | 0.445 |
| APS - BLIPS | -0.750 | -2.172 | 0.672 | 0.713 |
| APS - FEP | -0.726 | -2.362 | 0.909 | 0.808 |
| BLIPS - FEP | 0.024 | -1.950 | 1.998 | 1 |
| **Hippocampus** |  |  |  |  |
| HC - APS | 0.581 | -1.414 | 2.576 | 0.936 |
| HC - BLIPS | -0.094 | -2.753 | 2.564 | 1 |
| HC - FEP | 0.428 | -2.385 | 3.241 | 0.99 |
| APS - BLIPS | -0.675 | -2.961 | 1.611 | 0.934 |
| APS - FEP | -0.153 | -2.782 | 2.477 | 0.999 |
| BLIPS - FEP | 0.522 | -2.652 | 3.697 | 0.987 |
| **Striatum** |  |  |  |  |
| HC - APS | 0.306 | -1.580 | 2.192 | 0.988 |
| HC - BLIPS | -0.114 | -2.626 | 2.399 | 1 |
| HC - FEP | -0.362 | -3.021 | 2.297 | 0.993 |
| APS - BLIPS | -0.420 | -2.581 | 1.741 | 0.98 |
| APS - FEP | -0.668 | -3.154 | 1.818 | 0.949 |
| BLIPS - FEP | -0.248 | -3.249 | 2.752 | 0.998 |

Abbreviations: APS, attenuated psychosis syndrome; BLIPS, brief limited intermittent psychosis syndrome; CBF, cerebral blood flow; CI, confidence interval; FEP, first episode psychosis; HC, healthy controls.

**Supplementary Table S7** Results of right-lateralised ROI general linear models (no covariates)

| **Contrast** | **Estimated marginal mean difference** | **Lower 95% CI** | **Upper 95% CI** | **p-value** |
| --- | --- | --- | --- | --- |
| **Frontal cortex** | | | | |
| HC - APS | 0.404 | -2.572 | 3.380 | 0.993 |
| HC - BLIPS | -0.230 | -4.208 | 3.747 | 0.999 |
| HC - FEP | 1.434 | -2.543 | 5.411 | 0.887 |
| APS - BLIPS | -0.635 | -4.064 | 2.795 | 0.982 |
| APS - FEP | 1.030 | -2.400 | 4.459 | 0.93 |
| BLIPS - FEP | 1.664 | -2.663 | 5.991 | 0.866 |
| **Hippocampus** | | | | |
| HC - APS | -0.611 | -3.673 | 2.451 | 0.978 |
| HC - BLIPS | -1.232 | -5.324 | 2.861 | 0.93 |
| HC - FEP | -0.572 | -4.665 | 3.520 | 0.992 |
| APS - BLIPS | -0.621 | -4.150 | 2.908 | 0.985 |
| APS - FEP | 0.039 | -3.490 | 3.568 | 1 |
| BLIPS - FEP | 0.659 | -3.793 | 5.112 | 0.991 |
| **Striatum** | | | | |
| HC - APS | -0.161 | -2.495 | 2.172 | 0.999 |
| HC - BLIPS | -0.776 | -3.895 | 2.342 | 0.959 |
| HC - FEP | 0.081 | -3.037 | 3.200 | 1 |
| APS - BLIPS | -0.615 | -3.304 | 2.074 | 0.967 |
| APS - FEP | 0.243 | -2.446 | 2.932 | 0.998 |
| BLIPS - FEP | 0.858 | -2.535 | 4.250 | 0.957 |

**Supplementary Table S8** Results of right-lateralised ROI general linear models with global CBF as a covariate

| **Contrast** | **Estimated marginal mean difference** | **Lower 95% CI** | **Upper 95% CI** | **p-value** |
| --- | --- | --- | --- | --- |
| **Frontal cortex** | | | | |
| HC - APS | 0.008 | -1.285 | 1.302 | 1 |
| HC - BLIPS | -0.150 | -1.878 | 1.579 | 0.998 |
| HC - FEP | -0.736 | -2.473 | 1.002 | 0.829 |
| APS - BLIPS | -0.158 | -1.649 | 1.333 | 0.997 |
| APS - FEP | -0.744 | -2.242 | 0.753 | 0.749 |
| BLIPS - FEP | -0.586 | -2.475 | 1.304 | 0.924 |
| **Hippocampus** | | | | |
| HC - APS | -0.944 | -3.022 | 1.134 | 0.797 |
| HC - BLIPS | -1.164 | -3.940 | 1.613 | 0.833 |
| HC - FEP | -2.397 | -5.188 | 0.394 | 0.312 |
| APS - BLIPS | -0.220 | -2.615 | 2.175 | 0.998 |
| APS - FEP | -1.453 | -3.859 | 0.952 | 0.617 |
| BLIPS - FEP | -1.233 | -4.268 | 1.802 | 0.846 |
| **Striatum** | | | | |
| HC - APS | -0.414 | -2.009 | 1.182 | 0.954 |
| HC - BLIPS | -0.725 | -2.856 | 1.406 | 0.903 |
| HC - FEP | -1.300 | -3.443 | 0.842 | 0.614 |
| APS - BLIPS | -0.311 | -2.150 | 1.527 | 0.986 |
| APS - FEP | -0.887 | -2.733 | 0.960 | 0.768 |
| BLIPS - FEP | -0.575 | -2.905 | 1.754 | 0.96 |

Abbreviations: APS, attenuated psychosis syndrome; BLIPS, brief limited intermittent psychosis syndrome; CBF, cerebral blood flow; CI, confidence interval; FEP, first episode psychosis; HC, healthy controls.

**Supplementary Table S9** Results of right-lateralised ROI general linear models with global CBF, smoking status, age and sex as covariates

| **Contrast** | **Estimated marginal mean difference** | **Lower 95% CI** | **Upper 95% CI** | **p-value** |
| --- | --- | --- | --- | --- |
| **Frontal cortex** | | | | |
| HC - APS | 0.016 | -1.305 | 1.336 | 1 |
| HC - BLIPS | -0.080 | -1.839 | 1.680 | 1 |
| HC - FEP | -0.678 | -2.540 | 1.184 | 0.884 |
| APS - BLIPS | -0.095 | -1.608 | 1.418 | 0.999 |
| APS - FEP | -0.693 | -2.434 | 1.047 | 0.853 |
| BLIPS - FEP | -0.598 | -2.699 | 1.503 | 0.94 |
| **Hippocampus** | | | | |
| HC - APS | -0.854 | -2.965 | 1.257 | 0.847 |
| HC - BLIPS | -0.898 | -3.711 | 1.914 | 0.918 |
| HC - FEP | -2.971 | -5.947 | 0.005 | 0.189 |
| APS - BLIPS | -0.044 | -2.463 | 2.375 | 1 |
| APS - FEP | -2.116 | -4.899 | 0.666 | 0.421 |
| BLIPS - FEP | -2.072 | -5.431 | 1.286 | 0.601 |
| **Striatum** | | | | |
| HC - APS | -0.442 | -2.057 | 1.172 | 0.946 |
| HC - BLIPS | -0.922 | -3.073 | 1.229 | 0.824 |
| HC - FEP | -1.145 | -3.420 | 1.131 | 0.742 |
| APS - BLIPS | -0.480 | -2.329 | 1.370 | 0.954 |
| APS - FEP | -0.702 | -2.830 | 1.425 | 0.91 |
| BLIPS - FEP | -0.223 | -2.791 | 2.346 | 0.998 |

Abbreviations: APS, attenuated psychosis syndrome; BLIPS, brief limited intermittent psychosis syndrome; CBF, cerebral blood flow; CI, confidence interval; FEP, first episode psychosis; HC, healthy controls.

**Supplementary Table S11** Results of ROI general linear models excluding subjects with prior antipsychotic exposure

| **Contrast** | **Estimated marginal mean difference** | **Lower 95% CI** | **Upper 95% CI** | **p-value** |
| --- | --- | --- | --- | --- |
| **Global CBF** |  |  |  |  |
| HC - APS | 0.204 | -2.842 | 3.249 | 0.999 |
| HC - BLIPS | 0.313 | -3.731 | 4.357 | 0.999 |
| HC - FEP | 2.931 | -2.542 | 8.403 | 0.703 |
| APS - BLIPS | 0.109 | -3.452 | 3.671 | 1 |
| APS - FEP | 2.727 | -2.399 | 7.853 | 0.708 |
| BLIPS - FEP | 2.618 | -3.158 | 8.393 | 0.798 |
| **Frontal cortex** | | | | |
| HC - APS | 0.219 | -2.852 | 3.289 | 0.999 |
| HC - BLIPS | 0.295 | -3.782 | 4.372 | 0.999 |
| HC - FEP | 1.439 | -4.077 | 6.955 | 0.953 |
| APS - BLIPS | 0.077 | -3.514 | 3.667 | 1 |
| APS - FEP | 1.221 | -3.946 | 6.388 | 0.964 |
| BLIPS - FEP | 1.144 | -4.678 | 6.966 | 0.979 |
| **Hippocampus** | | | | |
| HC - APS | 0.031 | -2.718 | 2.781 | 1 |
| HC - BLIPS | -0.304 | -3.955 | 3.347 | 0.998 |
| HC - FEP | 0.846 | -4.093 | 5.786 | 0.986 |
| APS - BLIPS | -0.336 | -3.550 | 2.879 | 0.997 |
| APS - FEP | 0.815 | -3.812 | 5.442 | 0.985 |
| BLIPS - FEP | 1.150 | -4.063 | 6.363 | 0.971 |
| **Striatum** | | | | |
| HC - APS | 0.444 | -2.019 | 2.908 | 0.984 |
| HC - BLIPS | 0.100 | -3.171 | 3.371 | 1 |
| HC - FEP | 1.478 | -2.948 | 5.904 | 0.907 |
| APS - BLIPS | -0.344 | -3.224 | 2.537 | 0.995 |
| APS - FEP | 1.034 | -3.112 | 5.179 | 0.958 |
| BLIPS - FEP | 1.378 | -3.293 | 6.048 | 0.934 |
| **Frontal cortex (left)** | | | | |
| HC - APS | -0.223 | -3.396 | 2.951 | 0.999 |
| HC - BLIPS | -0.886 | -5.100 | 3.328 | 0.974 |
| HC - FEP | 1.481 | -4.221 | 7.183 | 0.953 |
| APS - BLIPS | -0.664 | -4.375 | 3.047 | 0.984 |
| APS - FEP | 1.704 | -3.637 | 7.044 | 0.918 |
| BLIPS - FEP | 2.367 | -3.650 | 8.385 | 0.858 |
| **Hippocampus (left)** | | | | |
| HC - APS | 0.844 | -1.863 | 3.550 | 0.923 |
| HC - BLIPS | 0.519 | -3.075 | 4.113 | 0.991 |
| HC - FEP | 2.080 | -2.783 | 6.944 | 0.825 |
| APS - BLIPS | -0.325 | -3.490 | 2.841 | 0.997 |
| APS - FEP | 1.237 | -3.319 | 5.792 | 0.947 |
| BLIPS - FEP | 1.561 | -3.571 | 6.694 | 0.928 |
| **Striatum (left)** | | | | |
| HC - APS | 0.681 | -1.978 | 3.341 | 0.955 |
| HC - BLIPS | 0.464 | -3.067 | 3.995 | 0.993 |
| HC - FEP | 1.160 | -3.618 | 5.938 | 0.961 |
| APS - BLIPS | -0.218 | -3.327 | 2.892 | 0.999 |
| APS - FEP | 0.478 | -3.997 | 4.954 | 0.996 |
| BLIPS - FEP | 0.696 | -4.346 | 5.738 | 0.992 |
| **Frontal cortex (right)** | | | | |
| HC - APS | 0.065 | -2.887 | 3.018 | 1 |
| HC - BLIPS | 0.007 | -3.914 | 3.927 | 1 |
| HC - FEP | 2.284 | -3.020 | 7.589 | 0.822 |
| APS - BLIPS | -0.058 | -3.511 | 3.394 | 1 |
| APS - FEP | 2.219 | -2.749 | 7.188 | 0.805 |
| BLIPS - FEP | 2.278 | -3.320 | 7.876 | 0.845 |
| **Hippocampus (right)** | | | | |
| HC - APS | -1.000 | -3.978 | 1.978 | 0.906 |
| HC - BLIPS | -1.115 | -5.069 | 2.840 | 0.941 |
| HC - FEP | 0.032 | -5.319 | 5.383 | 1 |
| APS - BLIPS | -0.115 | -3.597 | 3.368 | 1 |
| APS - FEP | 1.032 | -3.980 | 6.044 | 0.976 |
| BLIPS - FEP | 1.146 | -4.501 | 6.794 | 0.977 |
| **Striatum (right)** | | | | |
| HC - APS | -0.187 | -2.578 | 2.204 | 0.999 |
| HC - BLIPS | -0.528 | -3.703 | 2.647 | 0.987 |
| HC - FEP | 0.597 | -3.699 | 4.893 | 0.992 |
| APS - BLIPS | -0.341 | -3.137 | 2.455 | 0.995 |
| APS - FEP | 0.784 | -3.240 | 4.808 | 0.979 |
| BLIPS - FEP | 1.125 | -3.409 | 5.659 | 0.959 |

**Supplementary Table S12** Results of ROI general linear models excluding subjects with prior antidepressant exposure

| **Contrast** | **Estimated marginal mean difference** | **Lower 95% CI** | **Upper 95% CI** | **p-value** |
| --- | --- | --- | --- | --- |
| **Global CBF** |  |  |  |  |
| HC - APS | 0.838 | -2.375 | 4.051 | 0.953 |
| HC - BLIPS | 1.040 | -2.933 | 5.013 | 0.952 |
| HC - FEP | 2.314 | -1.594 | 6.222 | 0.633 |
| APS - BLIPS | 0.202 | -3.542 | 3.946 | 1 |
| APS - FEP | 1.476 | -2.199 | 5.151 | 0.85 |
| BLIPS - FEP | 1.274 | -3.081 | 5.629 | 0.935 |
| **Frontal cortex** | | | | |
| HC - APS | 1.203 | -2.173 | 4.578 | 0.89 |
| HC - BLIPS | 0.861 | -3.313 | 5.035 | 0.976 |
| HC - FEP | 1.454 | -2.652 | 5.560 | 0.892 |
| APS - BLIPS | -0.342 | -4.275 | 3.591 | 0.998 |
| APS - FEP | 0.251 | -3.610 | 4.112 | 0.999 |
| BLIPS - FEP | 0.593 | -3.982 | 5.169 | 0.994 |
| **Hippocampus** | | | | |
| HC - APS | -0.254 | -3.350 | 2.841 | 0.998 |
| HC - BLIPS | 0.257 | -3.572 | 4.085 | 0.999 |
| HC - FEP | 0.378 | -3.388 | 4.143 | 0.997 |
| APS - BLIPS | 0.511 | -3.096 | 4.118 | 0.992 |
| APS - FEP | 0.632 | -2.909 | 4.173 | 0.984 |
| BLIPS - FEP | 0.121 | -4.075 | 4.317 | 1 |
| **Striatum** | | | | |
| HC - APS | 0.377 | -2.281 | 3.036 | 0.992 |
| HC - BLIPS | 0.801 | -2.486 | 4.089 | 0.961 |
| HC - FEP | 0.925 | -2.309 | 4.159 | 0.939 |
| APS - BLIPS | 0.424 | -2.674 | 3.522 | 0.993 |
| APS - FEP | 0.548 | -2.493 | 3.588 | 0.984 |
| BLIPS - FEP | 0.124 | -3.480 | 3.727 | 1 |
| **Frontal cortex (left)** | | | | |
| HC - APS | 0.247 | -3.113 | 3.607 | 0.999 |
| HC - BLIPS | 0.130 | -4.025 | 4.285 | 1 |
| HC - FEP | 0.800 | -3.287 | 4.887 | 0.979 |
| APS - BLIPS | -0.117 | -4.032 | 3.799 | 1 |
| APS - FEP | 0.553 | -3.290 | 4.396 | 0.991 |
| BLIPS - FEP | 0.670 | -3.885 | 5.225 | 0.991 |
| **Hippocampus (left)** | | | | |
| HC - APS | 0.524 | -2.458 | 3.507 | 0.985 |
| HC - BLIPS | 0.576 | -3.112 | 4.265 | 0.989 |
| HC - FEP | 1.555 | -2.072 | 5.183 | 0.824 |
| APS - BLIPS | 0.052 | -3.423 | 3.528 | 1 |
| APS - FEP | 1.031 | -2.380 | 4.443 | 0.929 |
| BLIPS - FEP | 0.979 | -3.064 | 5.022 | 0.962 |
| **Striatum (left)** | | | | |
| HC - APS | 0.722 | -2.247 | 3.691 | 0.961 |
| HC - BLIPS | 1.050 | -2.622 | 4.722 | 0.939 |
| HC - FEP | 1.275 | -2.337 | 4.887 | 0.893 |
| APS - BLIPS | 0.328 | -3.132 | 3.788 | 0.998 |
| APS - FEP | 0.553 | -2.843 | 3.949 | 0.988 |
| BLIPS - FEP | 0.225 | -3.800 | 4.250 | 0.999 |
| **Frontal cortex (right)** | | | | |
| HC - APS | 0.662 | -2.476 | 3.800 | 0.974 |
| HC - BLIPS | 1.053 | -2.827 | 4.934 | 0.947 |
| HC - FEP | 1.262 | -2.555 | 5.079 | 0.91 |
| APS - BLIPS | 0.391 | -3.265 | 4.048 | 0.996 |
| APS - FEP | 0.600 | -2.989 | 4.190 | 0.987 |
| BLIPS - FEP | 0.209 | -4.045 | 4.463 | 1 |
| **Hippocampus (right)** | | | | |
| HC - APS | -1.096 | -4.561 | 2.370 | 0.92 |
| HC - BLIPS | 0.031 | -4.255 | 4.318 | 1 |
| HC - FEP | -0.418 | -4.634 | 3.798 | 0.997 |
| APS - BLIPS | 1.127 | -2.912 | 5.166 | 0.943 |
| APS - FEP | 0.678 | -3.287 | 4.642 | 0.986 |
| BLIPS - FEP | -0.450 | -5.148 | 4.249 | 0.997 |
| **Striatum (right)** | | | | |
| HC - APS | -0.118 | -2.542 | 2.307 | 1 |
| HC - BLIPS | 0.281 | -2.717 | 3.279 | 0.998 |
| HC - FEP | 0.098 | -2.851 | 3.047 | 1 |
| APS - BLIPS | 0.399 | -2.426 | 3.224 | 0.992 |
| APS - FEP | 0.216 | -2.557 | 2.989 | 0.999 |
| BLIPS - FEP | -0.183 | -3.469 | 3.104 | 0.999 |

**Supplementary References**

1. Allen, P. *et al.* Resting hyperperfusion of the hippocampus, midbrain, and basal ganglia in people at high risk for psychosis. *The American Journal of Psychiatry* **173**, 392–399 (2016).

2. Davies, C. *et al.* Oxytocin modulates hippocampal perfusion in people at clinical high risk for psychosis. *Neuropsychopharmacology* **44**, 1300–1309 (2019).

3. Thedens, D. R., Irarrazaval, P., Sachs, T. S., Meyer, C. H. & Nishimura, D. G. Fast magnetic resonance coronary angiography with a three-dimensional stack of spirals trajectory. *Magnetic Resonance in Medicine* **41**, 1170–1179 (1999).

4. Alsop, D. C. *et al.* Recommended implementation of arterial spin-labeled perfusion MRI for clinical applications: A consensus of the ISMRM perfusion study group and the European consortium for ASL in dementia. *Magnetic Resonance in Medicine* **73**, 102–116 (2015).

5. Selvaggi, P. *et al.* Reduced cortical cerebral blood flow in antipsychotic-free first-episode psychosis and relationship to treatment response. *Psychol. Med.* 1–11 (2022) doi:10.1017/S0033291722002288.
